# Investigation of Buckling and Failure in Thin-Walled Columns Fabricated from PLA and PETG Using FDM 3D Printing

**DOI:** 10.3390/ma18143346

**Published:** 2025-07-17

**Authors:** Denys Baranovskyi, Pawel Wysmulski, Patryk Rozylo, Hubert Debski, Maryna Bulakh, Marcin Kopyść, Sergey Myamlin

**Affiliations:** 1 Faculty of Mechanics and Technology, Rzeszow University of Technology, 4 Kwiatkowskiego Street, 37-450 Stalowa Wola, Poland; m.bulakh@prz.edu.pl; 2 Faculty of Mechanical Engineering, Lublin University of Technology, Nadbystrzycka 36, 20-618 Lublin, Poland; p.wysmulski@pollub.pl (P.W.); p.rozylo@pollub.pl (P.R.); h.debski@pollub.pl (H.D.); 3 Motonita Styx Corporation, 14/18 Żwirki i Wigury Street, 37-450 Stalowa Wola, Poland; mkopysc@gmail.com; 4 Department of Wagon Engineering and Product Quality, Ukrainian State University of Railway Transport, Feuerbakh sq. 7, 61050 Kharkiv, Ukraine; sergeymyamlin91@gmail.com

**Keywords:** thin-walled columns, buckling, failure, PLA, PETG, FDM printing, critical load

## Abstract

This paper presents the results of an experimental study on the buckling and failure behavior of thin-walled square columns made from PLA and PETG polymers using FDM 3D printing technology. Thin-walled square columns made from thermoplastic materials, intended for use in lightweight load-bearing applications such as structural supports in transportation, construction, and mechanical assemblies, were tested under axial compression from the onset of buckling to complete failure. The novelty of this work lies in the application of an interdisciplinary experimental approach to the analysis of the behavior of thin-walled columns made of PLA and PETG materials during FDM 3D printing under compression until complete failure, as well as the use of acoustic and optical diagnostic methods for a comprehensive assessment of damage. The experimental results are as follows: Buckling load (N): PLA—1175 ± 32, PETG1—1910 ± 34, PETG2—1315 ± 27. Ultimate load (N): PLA—2770, PETG1—4077, PETG2—2847. Maximum strain: PLA—11.35%, PETG1—11.77%, PETG2—10.99%. Among the tested materials, PETG1 exhibited the highest resistance and energy absorption capacity upon failure, making it a favorable choice for manufacturing 3D-printed load-bearing columns.

## 1. Introduction

Thin-walled load-bearing columns made of structural materials represent a unique class of structures characterized by high strength-to-weight ratios. These elements are commonly used in the form of thin-walled struts with either open or closed cross-sections [1]. While traditionally manufactured from metals—particularly aluminum alloys due to their high strength-to-weight ratio and excellent corrosion resistance—thin-walled columns are widely used in aerospace, automotive, and civil engineering applications where lightweight structural efficiency is essential. In aerospace engineering, for instance, thin-walled aluminium and composite cylindrical shells are used in fuselage sections, fuel tanks, and structural frames, where resistance to buckling under axial and combined loads is critical [2,3]. These studies underscore the continued relevance of thin-walled metallic structures and offer a foundation for comparison with polymer-based alternatives fabricated via additive manufacturing. Of particular interest are thin-walled load-bearing columns with closed cross-sections, which are designed to withstand axial loads, including post-buckling behavior—a phenomenon known as local or global buckling [4,5]. This instability, marked by a sudden change in deformation shape, remains a topic of significant scientific interest, especially since many composite elements retain their load-bearing capacity even after reaching a critical state.

However, the fabrication of thin-walled composite columns poses challenges due to high manufacturing time and costs. A promising solution involves the use of 3D printing with relatively simple polymer materials. Polymers have become central in various industries due to their light weight, high strength, chemical resistance, moldability, and cost-effectiveness [5,6]. The global production of polymers continues to rise, underlining their importance in the global economy. Their diversity enables wide-ranging applications: in mechanical engineering, plastics are used in structural components, insulation, friction parts, and high-strength composites [7,8]; in construction, they serve as pipes, window frames, insulation panels, and reinforcement elements [9,10]; and in the automotive and aerospace industries, the use of plastics in structural elements significantly reduces vehicle weight, fuel consumption, and CO_2_ emissions [8,11,12]. In medicine, plastics range from sterile packaging and disposable syringes to implants and biocompatible materials for regenerative therapies [13].

For 3D printing of thin-walled supporting columns, materials like polylactic acid (PLA) and polyethylene terephthalate glycol-modified (PETG) are viable options. PLA, as a biopolymer, is biodegradable and offers a low carbon footprint [14,15], while PETG is known for its high impact resistance and durability in aggressive environments, making it more suitable for technical and semi-industrial applications [16]. The use of additive manufacturing (FDM printing) for fabricating these columns enables the customization of product parameters, reduces tooling costs, and accelerates the production cycle [17].

Moreover, thin-walled columns made from PLA and PETG exhibit ease of processing and high reproducibility during FDM printing, making them attractive from both technical and economic perspectives.

## 2. Literature Review

In studies [13,14], it is stated that PLA and PETG represent two opposite ends of the modern polymer spectrum: PLA is biodegradable and derived from renewable resources, while PETG is petroleum-based and resistant to degradation. With increasing attention to sustainability, PLA has become a focus of active research [18]. Its physical and mechanical properties have been extensively studied and characterized in works [12,15,18]. However, PLA remains brittle, is sensitive to thermal degradation, and has limited fire resistance [6,19].

PETG, by contrast, exhibits high heat resistance and impact strength, although it decomposes poorly. In comparative studies with PLA [20], PETG demonstrates superior tensile strength and thermal stability. Efforts to improve the mechanical properties of PLA have been reported in numerous works [21,22,23,24]. These include the addition of natural fibers such as jute [21], okra [23], rice straw, and husk [22], as well as synthetic fillers like polyester and carbon fibers [24], which enhance the strength, stiffness, and impact resistance of PLA-printed parts. Studies also show that careful control of extrusion and annealing conditions improves PLA’s chemical and thermal resistance [6,25].

The properties of PLA/PS blends have been explored in [26], where the best compatibility was achieved at 50–75% PLA. Although PLA and PETG are typically incompatible due to phase separation, study [27] demonstrated that the use of compatibilizers like E-BA-GMA can improve both impact strength and heat resistance.

The influence of FDM printing parameters on the properties of PLA and PETG has been widely studied. For example, ref. [28] provides a detailed analysis of how print temperature, speed, and layer thickness affect thermal and mechanical characteristics. The main findings from [28] include the following:

- PLA exhibits higher tensile strength;
- PETG surpasses PLA in impact strength and heat resistance;
- Higher nozzle temperatures and lower layer heights improve strength and interlayer adhesion for both materials;
- PETG retains dimensional stability better at elevated temperatures.

Based on these findings, PETG is considered the preferred material for fabricating thin-walled load-bearing columns. However, studies [16,17,29,30,31] also highlight PLA’s advantages, particularly its low shrinkage and excellent printability. Optimization of printing parameters—such as layer thickness, nozzle speed, and infill density—significantly influences mechanical strength [29,30,31]. Additional research [5,30] demonstrates how parameter combinations can enhance material performance and reduce failure risk. Furthermore, incorporating microcrystalline cellulose improves wear resistance and mechanical strength in FDM-printed parts [32].

Study [33] examined the behavior of a PLA/PBAT blend under radiation exposure, emphasizing the relevance of such testing for assessing the long-term durability of biodegradable materials. In [34], the interaction of PLA with the alkaline environment of concrete was analyzed, revealing a decrease in strength after prolonged exposure—highlighting a key limitation for construction applications. The study underscores the necessity of modifying PLA to improve environmental resistance.

A review article [35] summarizes the advantages of PLA as a biodegradable polymer and discusses its physical and mechanical limitations. It also addresses future prospects for PLA enhancement through compositing and chemical modification. Study [36] investigates the effect of biocompatible polyelectrolytes on PLA/PBS composites, showing improved heat resistance and reduced flammability—offering an environmentally friendly strategy for material improvement in construction and packaging. Review [37] focuses on sustainable use of PLA-based biocomposites, emphasizing their potential across packaging, biomedical, and 3D printing applications.

To improve the mechanical performance of thermoplastic thin-walled structures fabricated via FDM, researchers have increasingly focused on reinforcement strategies and process optimization [38,39]. One promising direction involves the use of fiber-reinforced composites and recycled materials within the FDM workflow. A recent study on composite upcycling demonstrated that injection-molded parts made from recycled material extrusion-printed components containing glass fiber-reinforced polypropylene retained excellent mechanical performance, suggesting the feasibility of closed-loop recycling for structural parts in engineering applications [38]. Furthermore, a comprehensive literature review by the [39] highlights the mechanical advantages of sustainable polymer-based composites, including enhanced tensile strength, stiffness, and durability when reinforced with natural or synthetic fibers such as carbon, glass, and jute. The review also emphasizes the importance of printing parameters—including nozzle temperature, layer orientation, and infill density—in achieving optimal performance. In this context, future studies should examine the integration of recycled composite feedstock or continuous fiber reinforcement into FDM processes, as well as multi-material printing and in situ monitoring, to enhance structural integrity and sustainability. These developments could significantly expand the range of load-bearing applications for thin-walled thermoplastic columns in both technical and industrial domains.

The literature analyzed above underscores the importance of PLA as an eco-friendly polymer with broad applicability, and of PETG as a durable, recyclable material with long-term environmental stability. Current research continues to focus on enhancing thermal and chemical resistance, mechanical strength, and environmental durability.

This literature review has revealed several research gaps. Chief among them is the limited number of studies involving real components, products, or prototypes, as well as a lack of practical applications. In response, this study proposes the fabrication of thin-walled load-bearing columns using PLA and PETG through FDM 3D printing.

The aim of this work is to investigate the buckling and failure behavior of thin-walled columns made from PLA and PETG materials using FDM 3D printing technology.

The novelty of this study lies in the application of an interdisciplinary experimental approach to evaluate the compressive behavior of PLA and PETG thin-walled columns fabricated via FDM 3D printing, up to the point of complete failure. This includes the integration of acoustic and optical diagnostic techniques for comprehensive damage assessment.

## 3. Materials and Methods

### 3.1. Three-Dimensional Model

**Figure 1:**
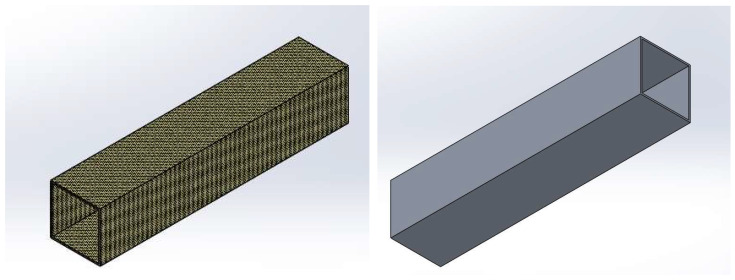
General view of a 3D model of a thin-walled column with a square cross-section measuring 40 mm × 40 mm, 200 mm long, and a wall thickness of 1.24 mm.

The 3D model was developed based on the practical application of thin-walled columns with a square cross-section subjected to axial compression. A general view of the 3D model of the thin-walled square columns is shown in Figure 1.

The thin-walled columns had a square cross-section with dimensions of 40 mm × 40 mm × 200 mm and a wall thickness of 1.24 mm. This wall thickness was chosen to allow for comparison with composite columns. The dimensions of the columns were selected during preliminary research carried out as part of the National Science Centre (Poland) project No. 2021/41/B/ST8/00148. Based on preliminary numerical simulations and experimental tests, it was estimated that the parameters of a 40 mm × 40 mm column cross-section with a height of 200 mm allow for the full number of half-waves to be obtained within the buckling of the structure. Many publications, for example, refs. [1,4,5], outline the results of stability and load-bearing capacity of thin-walled composite structures under axial compression. Tests on composite materials, conducted as part of an ongoing project with the National Science Centre (Poland), provided the basis for the current research on printed profiles with identical cross-sections. The dimensions of the specimens were selected to ensure that the full number of half-waves was obtained within the buckling range (without the jump effect), and the ratio of the profile height to width is 5/1, where the profile still indicates adequate slenderness. In the case of composite materials in the studies referred to above, several types of cross-sections with a uniform height of 200 mm were tested, while for the tests in this publication, columns manufactured using 3D printing technology were used for a reliable comparison of behavior with previously tested composite materials with identical dimensions. Based on all preliminary tests, it was observed that the selected parameters ensure the proper stability of the structure.

However, it should be noted that the testing methodology applied here differs from the conventional approach defined in ASTM E9-19 [40] and ASTM D695-15 [41]. While the ASTM standards typically employ solid or short-length specimens with well-defined support conditions and strain measurement methods (such as bonded strain gauges or extensometers), the present study uses additively manufactured hollow square columns, tested under axial compression using optical strain measurement (Aramis 2D, ZEISS Industrial Quality Solutions, Oberkochen, Germany) and acoustic emission techniques. These advanced diagnostics allow for full-field strain visualization and real-time crack detection, which are not covered in standard ASTM compression tests. Moreover, the geometry here emphasizes thin-walled instability and buckling behavior, aspects not thoroughly addressed by ASTM D695 or E9 [40,41], which are focused on compressive strength rather than post-buckling failure modes.

### 3.2. Three-Dimensional Printing Machine, Printing Materials and Test Specimens

In this work, the Original Prusa i3 MK3S 3D printer (Prusa Research, Prague, Czech Republic) was used to print thin-walled columns of square cross-section 40 mm × 40 mm and 200 mm long.

The thin-walled columns were printed vertically (height along the *Z* axis). This made it possible to minimize the area of the 3D printer table and save printing time. A frame was provided around the 3D model for good adhesion of the printed specimens to the table. A G-code was developed for printing thin-walled columns.

To ensure consistent fabrication of the test specimens, the printing parameters were carefully controlled and are summarized in Table 1 below.

All thin-walled column specimens were printed in a vertical orientation, with the 200 mm height aligned along the *Z*-axis, perpendicular to the build plate. This configuration was chosen to replicate realistic loading conditions under axial compression and to minimize anisotropy effects in the loading direction. Printing vertically also allowed for efficient use of the build area and reduced warping by ensuring uniform layer deposition. However, this orientation can result in weaker inter-layer adhesion compared to in-plane loading, making it particularly relevant for studying the buckling and failure characteristics of FDM-printed structures under realistic stress scenarios.

**Figure 2:**
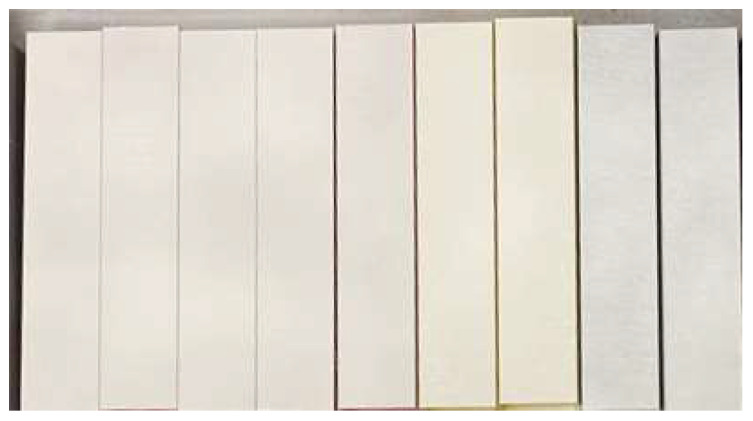
Test specimens of thin-walled columns obtained on a 3D printer from PLA and PETG materials.

After printing on a 3D printer, three test specimens of thin-walled columns were obtained (Figure 2).

Figure 2 shows the specimens after applying white paint. For the study on the Aramis 2D optical strain measurement system, it was necessary to create a mesh to record the deformation. After applying the white paint, graphite dots were applied to create a mesh.

The calculated mass (G-code) and measured mass of the obtained test specimens on the 3D printer were as follows:

- PLA—45.17 ± 0.14 g.
- PETG—46.34 ± 0.17 g.

### 3.3. Equipment Used for Research

**Figure 3:**
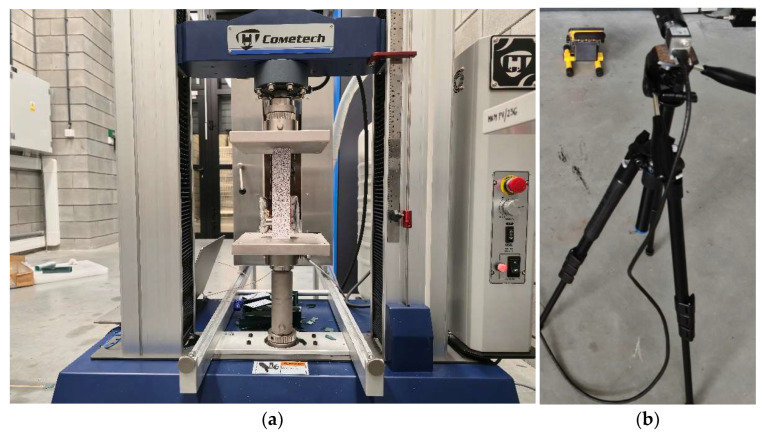
Cometech QC-505M2F universal testing machine with installed test specimen and VS150-L piezoelectric sensor (**a**) and Aramis 2D optical strain measurement system (**b**).

To study the deformation and failure of thin-walled columns manufactured using 3D printing from PLA and PETG materials, the following equipment was used:

- Cometech QC-505M2F universal testing machine (Cometech Testing Machines Co., Ltd., Fengyuan District, Taichung City, Taiwan) (Figure 3a). This machine is designed to perform static compression tests; it is controlled by Amis Plus software (1.5.6); maximum load is 50 kN; initial load is 100 N; test speed is 4 mm/min.
- Aramis 2D optical strain measurement system (Figure 3b). This equipment is a digital image correlation (DIC) technology; it provides visualization of strain distribution over the specimen surface and allows to determine zones with maximum stress and potential damage.
- SpotWave 201 acoustic emission system (Vallen Systeme GmbH, Wolfratshausen, Germany). This equipment is presented as a single-channel system with a VS150-L piezoelectric sensor (Vallen Systeme GmbH, Wolfratshausen, Germany) (Figure 3a); it is used to record signals accompanying material damage. As a result, the obtained data were analyzed using Vallen AE software (R2023.1218.2).

Thus, to study the buckling and failure of thin-walled columns manufactured using 3D printing from PLA and PETG materials, a set of mechanical, acoustic, and optical methods was used, providing an interdisciplinary approach to the analysis of the destruction of the materials under study.

Using the above equipment, a comprehensive interdisciplinary experimental study was performed on the behavior of thin-walled columns made of PLA and PETG materials during FDM printing under compression until complete destruction. The results are presented below.

## 4. Results and Discussion

**Figure 4:**
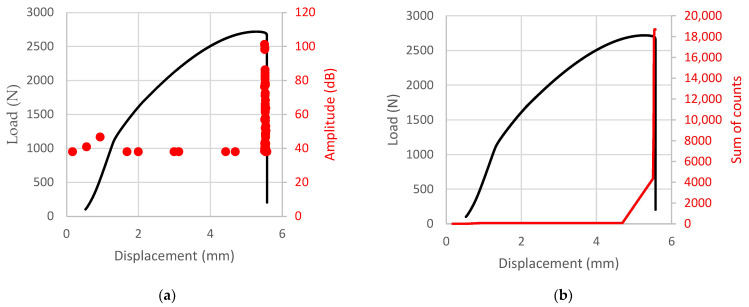

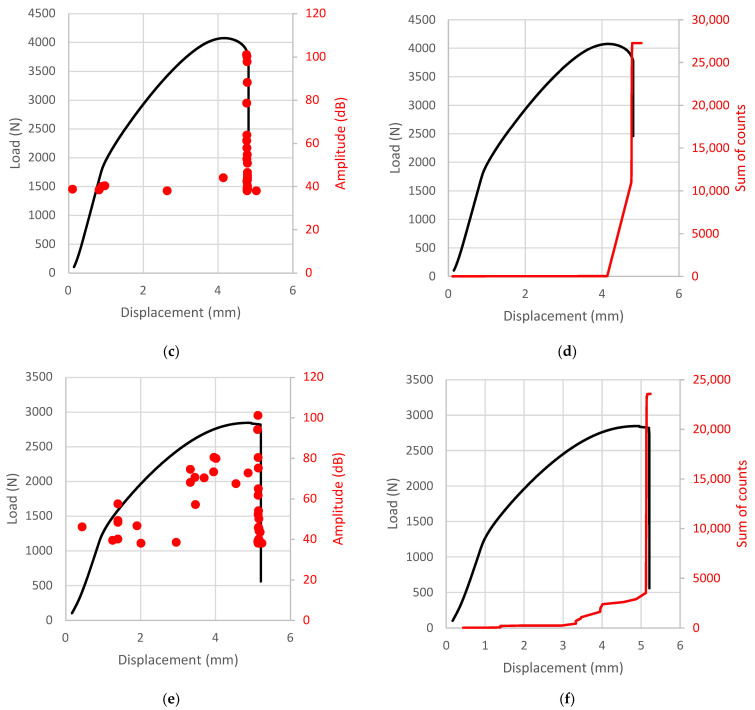
Load–displacement diagram with sound amplitude (**a**,**c**,**e**) and sum of counts of acoustic emission pulses (**b**,**d**,**f**) for the test specimen: PLA (**a**,**b**); PETG1 (**c**,**d**); PETG2 (**e**,**f**).

The results obtained on the Cometech QC-505M2F universal testing machine, recorded in the form of a load–displacement diagram and the sound amplitude from the piezoelectric sensor are shown in Figure 4.

The load–displacement curve (Figure 4a) for the PLA specimen shows a sharp increase in load, reaching a peak of approximately 2720 ± 41 N at a displacement of 5.3 ± 0.2 mm. At the end of the loading process, a sudden drop in load is observed, indicating a brittle failure mode, albeit with some evidence of plastic deformation. The onset of buckling occurs at a load of 1175 ± 32 N and a displacement of 1.4 ± 0.1 mm, up to which the PLA specimen remains geometrically stable. Acoustic emission (AE) data reveal a distinct set of sound amplitude peaks corresponding to the failure event. Notably, no significant amplitude peak is recorded at the buckling point; however, a noticeable drop in amplitude occurs at a displacement of 1.4 ± 0.1 mm. The AE pulse count data (Figure 4b), plotted against the corresponding displacements and loads, are in good agreement with the final failure of the PLA specimen.

For the PETG1 specimen (Figure 4c), the buckling point occurs at a load of 1910 ± 34 N and a displacement of 0.9 ± 0.1 mm, confirmed by the AE method, which shows multiple amplitude events in the 0.9–1.1 mm range. Prior to this point, the PETG1 specimen maintains geometric stability. The load–displacement curve displays a sharp increase in force, reaching a peak of approximately 4077 ± 44 N at 4.0 ± 0.1 mm of displacement. As the displacement increases to 4.5 ± 0.2 mm, the load decreases slightly to 3885 ± 41 N, indicating a high resistance to fracture. Final fracture occurs at approximately 4.6 mm, as confirmed by sound amplitude peaks in the 4.5–4.6 mm displacement range. The AE pulse count data (Figure 4d) also confirm the completion of the fracture process at 4.6 ± 0.2 mm.

Another PETG2 specimen (Figure 4e) exhibits a sharp increase in load up to a peak of 2847 ± 33 N at a displacement of approximately 4.9 mm. At the final loading stage, a sudden drop in load indicates brittle fracture, with noticeable signs of plastic deformation. Buckling is observed at a load of 1315 ± 27 N and a displacement of 1.0 ± 0.1 mm, with the material behaving stably up to this point. AE analysis shows a clear spectrum of sound amplitudes that align with the failure event at 5.2 ± 0.2 mm. While no amplitude peak is observed during the initial buckling (around 1.0 ± 0.1 mm), a sequence of increasing amplitudes is recorded leading up to final failure. The AE pulse count data (Figure 4f) correlate well with the displacement of 5.2 ± 0.2 mm, indicating the completion of the fracture process.

Furthermore, a clear correlation was observed between damage propagation in the thin-walled columns and the sum of counts of AE pulses. As shown in Figure 4b,d,f, the sharp increase in AE counts coincides with the onset of rapid or unstable crack growth, particularly in the final stages of loading. For example, in the PETG1 specimen (Figure 4d), a significant surge in AE activity is observed just before the ultimate load is reached, indicating the transition from stable deformation to catastrophic failure.

This pattern is consistent across all specimens and aligns with findings reported in other studies, where a sudden rise in AE count rates is widely recognized as an indicator of rapid crack propagation or unstable fracture events in polymeric and composite materials. The AE pulse accumulation reflects internal structural degradation and micro-crack coalescence, providing a non-destructive early warning of imminent failure. In this study, the combination of mechanical data and AE diagnostics provides a robust tool for monitoring the progression of damage in thermoplastic thin-walled columns fabricated by FDM.

**Figure 5:**
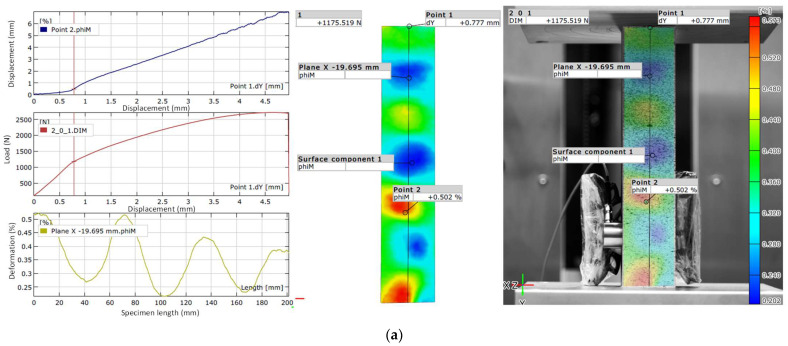

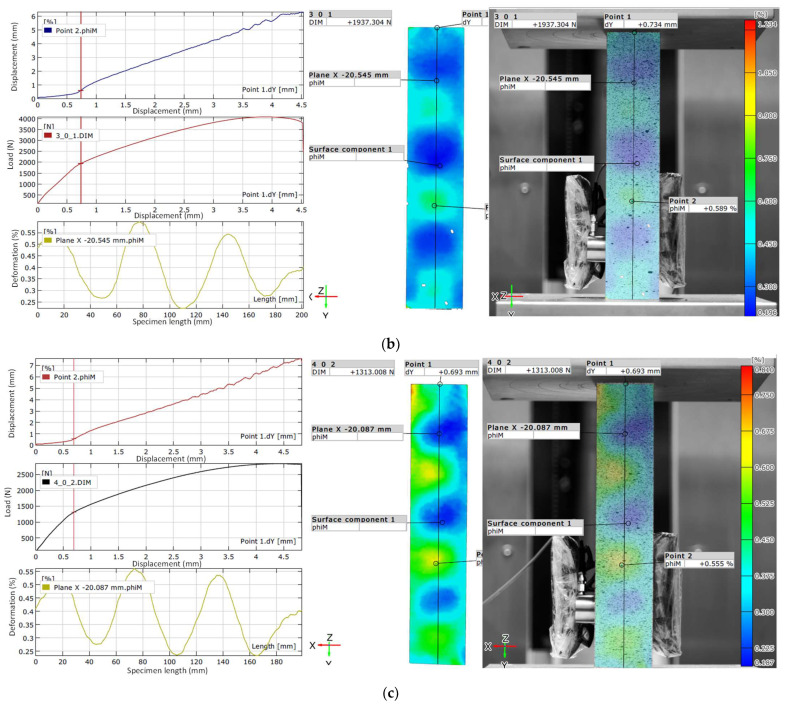
Experimental results of the onset of the buckling process of the test specimen, obtained using the Aramis 2D optical strain measurement system for the material: PLA (**a**); PETG1 (**b**); PETG2 (**c**).

The experimental results obtained using the Aramis 2D optical strain measurement system at the onset of buckling in the test specimens are presented in Figure 5.

On the left side of each panel in Figure 5 (and in all subsequent figures), three graphs are presented: the first shows the displacement of Point 2 as a function of Point 1 displacement; the second shows the load as a function of Point 1 displacement; and the third illustrates the strain distribution along the length of the specimen. On the right side, each subfigure includes a full-field strain map, obtained with the Aramis 2D optical measurement system, illustrating the spatial distribution of de-formation at the moment of fracture.

The presented results (Figure 5a) show that the PLA specimen enters a phase in which buckling remains reversible and can be controlled by reducing the applied load. A localized deformation zone is visible along the axis of symmetry, indicating a transition from linear deformation to geometric instability. The color map of the PLA specimen displays a smooth gradient, suggesting moderate material plasticity. Deformation localization begins at a relatively low displacement of 1.4 mm. At this point, Point 1 of the PLA specimen shifts by 0.777 mm. At Point 2, under a load of approximately 1176 N, a strain of 0.502% is recorded. The maximum strain associated with buckling in the PLA specimen reaches 0.573% under the same load. The distribution of deformation along the specimen length, visualized as multicolored regions, characterizes the onset of buckling. The wave-like deformation pattern, shown in the lower left graph, further confirms the buckling process and aligns with the results from the Aramis 2D optical deformation measurement system.

Figure 5b shows that the PETG1 specimen exhibits the most extensive and well-distributed buckling pattern under a load of 1900 N and a displacement of 0.9 mm. Deformation zones span a significant portion of the specimen’s length, demonstrating a high capacity for plastic deformation. A localized deformation zone forms along the axis of symmetry, marking the transition to geometric instability. The color zones are rounded with relatively sharp boundaries, suggesting a uniform stress distribution along both the length and width of the specimen. The multicolored regions indicate the onset of buckling. Deformation localization in the PETG1 specimen begins at a displacement of approximately 0.75 mm, with Point 1 shifting by 0.734 mm. At Point 2, under a load of about 1900 N, a strain of 0.589% is observed. The maximum strain at buckling onset reaches 1.234% under the same load. The wave-like deformation profile and the color map from the Aramis 2D system confirm the onset and progression of the buckling process.

As shown in Figure 5c, the PETG2 specimen displays a weak manifestation of the initial buckling stage. At a load of 1313 N and a displacement of 1.0 mm, the specimen shows the least pronounced and least evenly distributed buckling pattern. Deformation zones extend over a large portion of the specimen, indicating a considerable ability for plastic reshaping. The image reveals an asymmetric localized deformation zone, signaling a shift toward geometric instability. The blurred and indistinct color areas indicate an uneven stress distribution along the specimen’s length and width. Nonetheless, the multicolored regions still reflect the early phase of buckling. Deformation localization begins at a displacement of about 1.0 mm. At this point, Point 1 is displaced by 0.693 mm. At Point 2, under a load of approximately 1313 N, a strain of 0.555% is measured. The maximum strain in this initial buckling stage is 0.810% under the same load. The wave-like strain pattern along the specimen, shown in the lower left graph, provides a clearer representation of emerging buckling zones.

The PETG2 specimen demonstrates an earlier and more irregular onset of buckling compared to the PETG1 specimen. Although the PETG2 specimen sustains a higher buckling load than the PLA specimen, the PLA specimen exhibits a more clearly defined deformation pattern.

**Figure 6:**
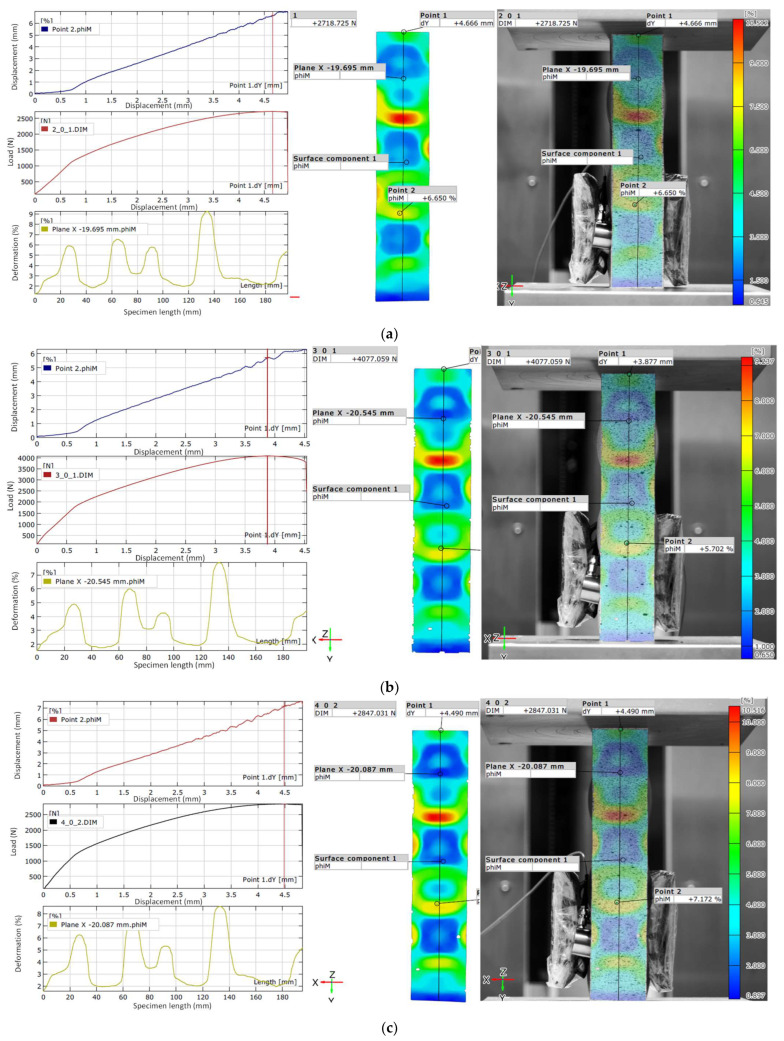
Experimental results at the point of ultimate load the test specimen obtained using the Aramis 2D optical strain measurement system for the material: PLA (**a**); PETG1 (**b**); PETG2 (**c**).

The experimental results, obtained at the ultimate load using the Aramis 2D optical strain measurement system, are shown in Figure 6.

The ultimate load for the PLA specimen (Figure 6a) is a constant 2719 N, with a displacement ranging from 5.24 to 5.26 mm. The maximum strain recorded is 10.512%. The deformation zone is clearly formed along the axis of symmetry. The strain distribution in the PLA specimen exhibits distinct deformation “islands” with well-defined borders, and the color map displays a smooth gradient transition. At Point 1, the PLA specimen experiences a displacement of 4.666 mm. At Point 2, under the same load of approximately 2719 N, the strain reaches 6.650%. The wave-like variation in strain along the length of the PLA specimen, shown in the graph on the left, corresponds closely with the color pattern recorded using the Aramis 2D optical deformation measurement system, presented below.

For the PETG1 specimen (Figure 6b), the ultimate load is a constant 4077 N, with a displacement of 4.14 to 4.16 mm. The maximum strain observed is 9.237%. The deformation zone also forms along the axis of symmetry. The color zones display clear, rounded shapes and sharp boundaries, indicating a uniform stress distribution across the length and width of the PETG1 specimen. At the ultimate load, Point 1 displaces by 3.877 mm, while at Point 2, under the same load of approximately 4077 N, the strain is recorded at 5.702%.

In the case of the PETG2 specimen (Figure 6c), the ultimate load is a constant 2847 N, with a displacement of 4.92 to 4.94 mm. The maximum strain reaches 10.516%. The deformation zone is distinctly aligned along the symmetry axis. The color map of the PETG2 specimen also features a gradient transition with clear boundaries. At Point 1, the displacement is 4.490 mm. At Point 2, under the same load of approximately 2847 N, a strain of 7.172% is observed. The wave-like pattern of deformation along the length of the PETG2 specimen, as shown in the graph on the lower left, closely mirrors the strain distribution captured using the Aramis 2D optical measurement system.

**Figure 7:**
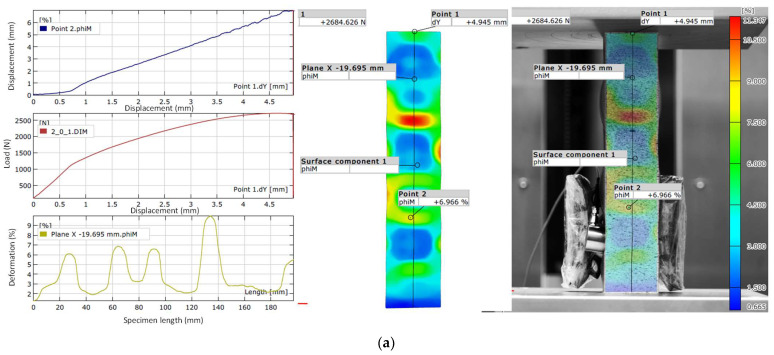

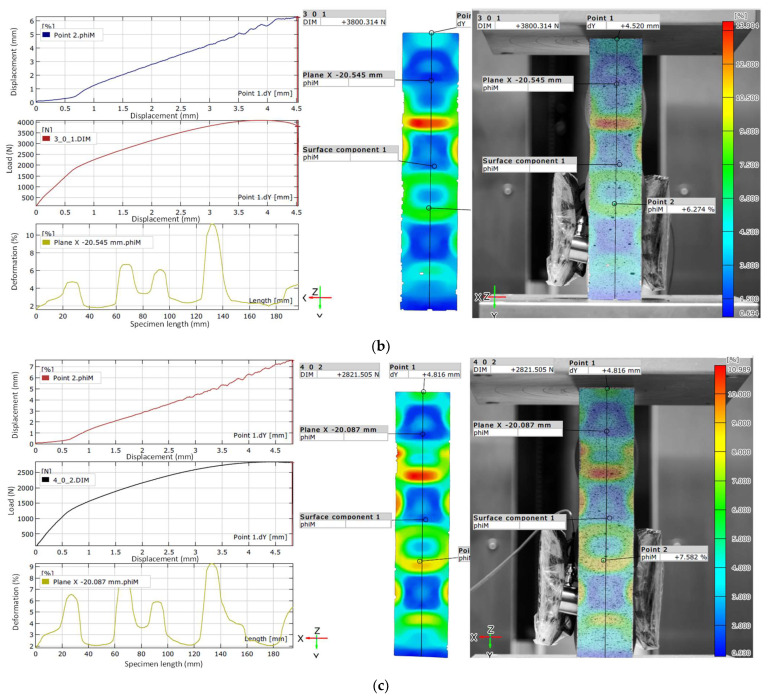
Experimental results obtained using the Aramis 2D optical deformation measurement system before failure for the material: PLA (**a**); PETG1 (**b**); PETG2 (**c**).

The experimental results obtained using the Aramis 2D optical deformation measurement system prior to failure are presented in Figure 7.

Based on the research results presented in Figure 7, failure occurs at the following values:

Failure loads for specimens:

- PLA—2684.6 N
- PETG1—3800.3 N
- PETG2—2821.5 N

Maximum displacement of specimens:

- PLA—5.1 mm
- PETG1—4.6 mm
- PETG2—5.2 mm

Maximum strain of specimens:

- PLA—11.347%
- PETG1—13.904%
- PETG2—10.989%

**Figure 8:**
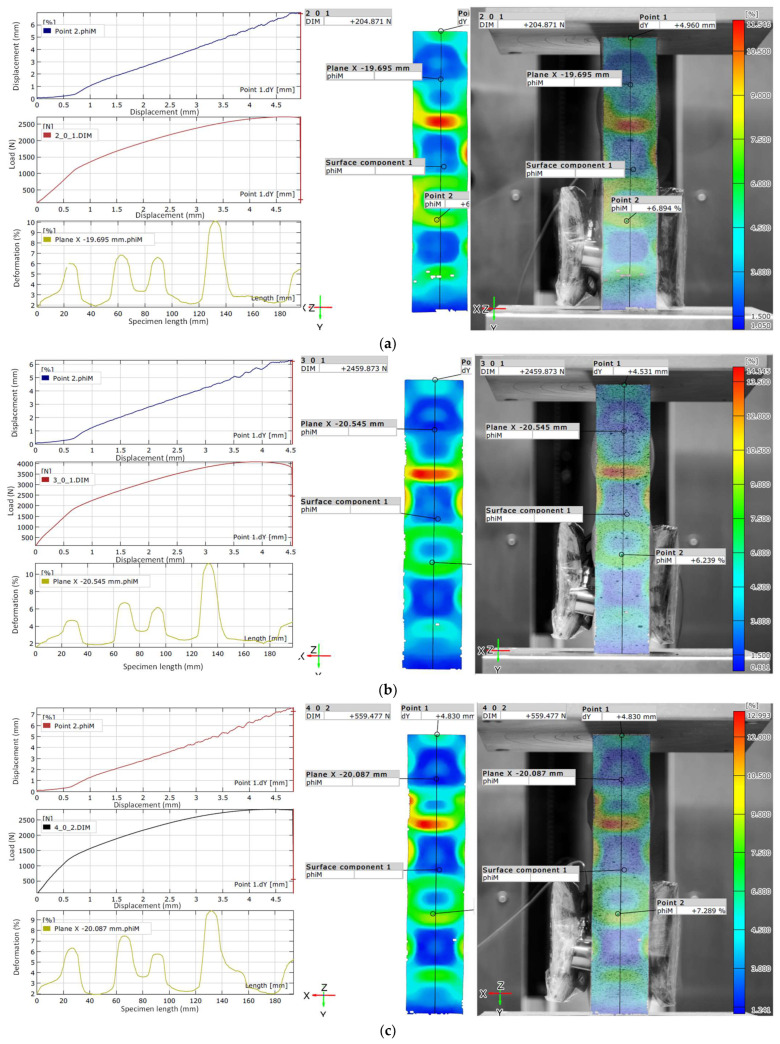
The failure moment of the test specimen, obtained using the Aramis 2D optical strain measurement system for the material: PLA (**a**); PETG1 (**b**); PETG2 (**c**).

The moment of failure for the test specimen, captured using the Aramis 2D optical strain measurement system, is shown in Figure 8.

Figure 8 presents the results obtained using the Aramis 2D system at the moment of failure of the test specimens made from three materials: PLA, PETG1, and PETG2. These images capture the final stage of failure—when the specimen physically fractures—and illustrate the behavior of the materials under critical deformation.

The failure mode and progression appear generally similar across all materials. However, the moment of failure observed in the PLA and PETG2 specimens is particularly alike in appearance.

On the left side of each panel, three graphs are shown:

The first graph represents the displacement of Point 2 versus Point 1, allowing assessment of vertical deformation behavior.

The second graph shows the applied load as a function of Point 1 displacement, illustrating the mechanical response just prior to failure.

The third graph presents the strain distribution along the specimen length, helping to localize the most deformed zones.

On the right side, each subfigure includes a full-field strain map, obtained with the Aramis 2D optical measurement system, illustrating the spatial distribution of deformation at the moment of fracture.

The observed values at the failure point were as follows:

PLA (Figure 8a): Final load: 204.9 N; Maximum displacement: 5.2 mm; Maximum strain: 11.546%; At Point 1: displacement ≈ 4.96 mm; At Point 2: strain ≈ 6.894%.

PETG1 (Figure 8b): Final load: 2459.9 N; Maximum displacement: 4.6 mm; Maximum strain: 14.145%; At Point 1: displacement ≈ 4.531 mm; At Point 2: strain ≈ 6.239%.

PETG2 (Figure 8c): Final load: 559.477 N; Maximum displacement: 5.2 mm; Maximum strain: 12.993%; At Point 1: displacement ≈ 4.830 mm; At Point 2: strain ≈ 7.289%.

The observed differences in failure point values among PLA, PETG1, and PETG2 can be attributed to a combination of material composition, microstructural characteristics, and geometrical or printing-induced imperfections. PETG1, which exhibited the highest final load and strain capacity, is likely characterized by a more uniform amorphous microstructure and better interlayer adhesion due to its optimized formulation and printing behavior.

In contrast, PLA, despite showing a comparable maximum displacement, failed at a much lower load (204.9 N), indicating its brittle nature and lower energy absorption. The relatively high strain value at failure (11.546%) may result from localized deformation just before catastrophic collapse, rather than from uniform plasticity.

PETG2, while mechanically superior to PLA, displayed a notably lower final load (559.5 N) than PETG1, possibly due to the presence of additives or fillers (e.g., carbon-based components), which can increase stiffness but introduce internal stress concentrators or compromise interlayer cohesion. Additionally, minor inconsistencies during FDM printing (e.g., slight variations in print speed, cooling rate, or filament quality) may also contribute to variability in mechanical performance, especially near the failure point.

Overall, the combination of material formulation and process-dependent factors explains the observed spread in load, displacement, and strain at failure, despite nominally identical specimen geometries and testing protocols.

It should be noted that slight differences in the appearance or number of plots between the subfigures may result from data capture resolution and the dynamic nature of the final failure process. However, all specimens were analyzed using an identical experimental procedure to ensure comparability.

These results demonstrate that PETG1 maintained the highest deformation capacity and load resistance at failure, confirming its superior mechanical performance among the tested materials.

According to material datasheets, the compressive strength of PLA and PETG1 and PETG2 is not always directly reported, as tensile properties are more commonly emphasized. However, based on available data and extrapolated values from tensile and flexural tests, typical compressive strengths range from 55 to 70 MPa for PLA, 60–70 MPa for PETG1, and 50–60 MPa for PETG2. In our study, the ultimate compressive load for PETG1 reached 4077 N, corresponding to a compressive stress (considering cross-sectional area) that fits within this expected range, though slightly lower due to geometric imperfections, 3D printing porosity, and stress concentration at buckling zones. This reduction is well-documented in previous studies on FDM-printed materials, where interlayer bonding and print orientation significantly influence strength performance.

The cross-sectional area the thin-walled column with a square cross-section measuring 40 mm × 40 mm, 200 mm long, and a wall thickness of 1.24 mm is approximately 192.25 mm^2^.

The calculated compressive strength values are summarized in Table 2.

For example, in [28] it was shown that PETG retains better dimensional stability and mechanical integrity at elevated temperatures compared to PLA, and that lower layer heights and higher nozzle temperatures improved interlayer adhesion. Their findings support our use of PETG1 under higher temperature and slower speed settings, which correlated with its superior mechanical behavior.

In a closely related study by [7] on 3D-printed PLA under compression, the specimens failed at compressive loads of approximately 2200–2700 N, comparable to the PLA failure load of 2720 N observed here. However, in [7], shorter solid cylinders were used, while our study employed thin-walled square columns, which are more sensitive to local buckling and geometric instability, further validating the robustness of our PLA specimen design despite inherent brittleness.

The work of [1] also conducted failure analysis of thin-walled composite columns and observed that local buckling modes initiated well before full failure, often without a dramatic drop in load. Similarly, in our PETG2 specimens, the onset of local buckling occurred at 1315 ± 27 N, yet the structure maintained load-bearing capacity up to 2847 N, demonstrating classic post-buckling stability. This is in contrast to PLA, where brittle collapse followed closely after buckling. Such behavior confirms the ductility advantage of PETG2, aligning well with other research emphasizing its superior strain tolerance and damage progression characteristics.

Importantly, our integration of AE analysis provides additional evidence supporting this behavior. Studies in structural health monitoring have shown that a sharp increase in AE event counts often precedes unstable crack growth (as described by [3]). The AE data in this study correlate well with the load–displacement curves and visual deformation fields, reinforcing the role of AE as an early-warning indicator for failure in polymer columns.

In conclusion, while datasheet values offer a baseline, they often reflect idealized, isotropic samples under quasi-static tensile loading. By contrast, our study replicates real-world conditions involving geometrically complex, anisotropic, and layered structures—thus bridging the gap between nominal material properties and functional performance in actual FDM-fabricated load-bearing components.

## 5. Conclusions

In this study, we conducted a comprehensive investigation into the buckling and failure behavior of thin-walled columns fabricated from PLA and PETG materials using FDM 3D printing technology. A combination of experimental techniques—including mechanical testing, optical digital image correlation (Aramis 2D system), and acoustic emission analysis—enabled us to obtain a detailed understanding of the mechanical response of the specimens under axial loading up to complete failure.

The results revealed that the two materials exhibit distinct deformation and failure mechanisms. PLA columns demonstrated a brittle fracture pattern characterized by minimal plastic deformation and an early onset of buckling. In contrast, PETG specimens—particularly those labeled PETG1—showed greater resistance to buckling, the ability to sustain higher loads, and significant deformation prior to failure. PETG2 exhibited intermediate behavior in terms of strength and stability, falling between PLA and PETG1.

By comparing data from the acoustic emission system with the deformation patterns visualized via Aramis 2D, we were able to precisely identify the onset of buckling, stress concentration zones, and failure initiation. The Aramis 2D system enabled visualization of strain distribution along the full length of the specimens and allowed us to track the development of localized deformation zones preceding failure.

The findings indicate that PETG, especially the PETG1 variant, offers the most favorable balance of strength and ductility for the fabrication of load-bearing elements via FDM printing. It demonstrated superior resistance to longitudinal deformation, maintained its geometric integrity until failure, and exhibited greater energy absorption compared to PLA.

The data obtained in this study can inform the design and development of thin-walled, load-bearing structures made from thermoplastics using additive manufacturing. These results also pave the way for further optimization of structural geometry, printing parameters, and material selection to enhance the strength and reliability of 3D-printed components.

### Prospects for Future Research

The results obtained confirm the feasibility of using PLA and PETG polymer materials to fabricate thin-walled load-bearing structures through FDM 3D printing. However, several aspects require further investigation and optimization. Future research will focus on the following key areas:

- Effect of Printing Parameters: A more detailed analysis will be conducted on how FDM printing parameters—such as extruder temperature, layer orientation, printing speed, and infill density—affect the mechanical properties and stability of thin-walled columns. This will support the development of recommendations for optimizing printing settings to maximize the strength and stability of the printed structures.
- Analysis of Geometries and Cross-Sections: Subsequent experiments will explore columns with various cross-sectional shapes, wall thicknesses, and element lengths to determine the most effective configurations for different loading conditions.
- Cyclic and Impact Loading: The performance of PLA and PETG structures under cyclic and dynamic loads will be examined. Investigating their fatigue resistance and impact toughness will help expand their practical applications, particularly in the transportation and construction sectors.
- Incorporation of Reinforcing Fillers: Future studies will explore the use of modified composite materials based on PLA and PETG, reinforced with fibers such as carbon, glass, or Kevlar. The influence of these fillers on critical load capacity and failure mechanisms will be a key focus.

## Figures and Tables

**Table 1:** FDM 3D printing parameters for thin-walled column specimens.

Parameter	PLA	PETG1	PETG2
Filament type	Print-Me PLA (PPHU POLIGRAF Wiesław Kasprowiak, under their PRINT-ME brand, Gorzów Wielkopolski, Poland)	Print-Me Swift PETG (PPHU POLIGRAF Wiesław Kasprowiak, under their PRINT-ME brand, Gorzów Wielkopolski, Poland)	ROSA 3D CarbonLook PET-G (ROSA PLAST Sp. Z o.o., Warsaw, Poland)
Filament diameter	1.75 mm	1.75 mm	1.75 mm
Nozzle temperature	~210 °C	~240 °C	~240 °C
Bed temperature	60 °C	80 °C	80 °C
Layer height	0.3 mm	0.3 mm	0.3 mm
Print speed	0.30 mm/s	0.30 mm/s	0.20 mm/s
Bed cooling	On	Off	Off
Printing orientation	Vertical (*Z*-axis)	Vertical (*Z*-axis)	Vertical (*Z*-axis)
Adhesion method	Framed base	Framed base	Framed base

**Table 2:** The calculated compressive strength values.

Material	Maximum Load (N)	Cross-Sectional Area (mm^2^)	Compressive Strength (MPa)
PLA	2720	192.25	14.15
PETG1	4077	192.25	21.21
PETG2	2847	192.25	14.81

## Data Availability

The original contributions presented in the study are included in the article, further inquiries can be directed to the corresponding author.
